# Deep Learning Convolutional Neural Network for the Retrieval of Land Surface Temperature from AMSR2 Data in China

**DOI:** 10.3390/s19132987

**Published:** 2019-07-06

**Authors:** Jiancan Tan, Nusseiba NourEldeen, Kebiao Mao, Jiancheng Shi, Zhaoliang Li, Tongren Xu, Zijin Yuan

**Affiliations:** 1 Institute of Agricultural Resources and Regional Planning, Chinese Academy of Agricultural Sciences, Beijing 100081, China; 2 College of Resources & Environment, Hunan Agricultural University, Changsha 410128, China; 3 State Key Laboratory of Remote Sensing Science, Institute of Remote Sensing and Digital Earth Research, Chinese Academy of Science and Beijing Normal University, Beijing 100101, China

**Keywords:** soil moisture, CNN, passive microwave remote sensing, LST retrieval

## Abstract

A convolutional neural network (CNN) algorithm was developed to retrieve the land surface temperature (LST) from Advanced Microwave Scanning Radiometer 2 (AMSR2) data in China. Reference data were selected using the Moderate Resolution Imaging Spectroradiometer (MODIS) LST product to overcome the problem related to the need for synchronous ground observation data. The AMSR2 brightness temperature (TB) data and MODIS surface temperature data were randomly divided into training and test datasets, and a CNN was constructed to simulate passive microwave radiation transmission to invert the surface temperature. The twelve V/H channel combinations (7.3, 10.65, 18.7, 23.8, 36.5, 89 GHz) resulted in the most stable and accurate CNN retrieval model. Vertical polarizations performed better than horizontal polarizations; however, because CNNs rely heavily on large amounts of data, the combination of vertical and horizontal polarizations performed better than a single polarization. The retrievals in different regions indicated that the CNN accuracy was highest over large bare land areas. A comparison of the retrieval results with ground measurement data from meteorological stations yielded R^2^ = 0.987, RMSE = 2.69 K, and an average relative error of 2.57 K, which indicated that the accuracy of the CNN LST retrieval algorithm was high and the retrieval results can be applied to long-term LST sequence analysis in China.

## 1. Introduction

Land surface temperature (LST) is an important factor for measuring the energy balance of Earth’s surface. LST is one of the most influential factors in climate, ecology and agriculture [1]. The timely and effective acquisition of large-scale LST information is of great practical significance for regional climate and agricultural monitoring [2,3,4]. As the most effective method to obtain land surface information over large areas, remote sensing can rapidly acquire LST information. After years of research and development, thermal infrared LST retrieval algorithms, including MODIS, and AVHRR data, have been developed and achieved high precision [5,6,7,8,9]. However, thermal infrared remote sensing is greatly influenced by moisture in the atmosphere and cannot penetrate the cloud layer, making it difficult to apply in all weather conditions [10,11]. Previous researchers analyzed the thermal infrared LST products provided by NASA, and most of the LST products in 60% of the middle and low latitude areas were affected by clouds and missing data, which resulted in certain limitations to practical applications [12,13]. Moreover, passive microwave satellite sensors have the advantages of being applicable in all weather conditions, can quickly achieve global coverage and are conducive to the study of the temporal and spatial variations in LST over large regional scales [14,15,16]. Therefore, how to use passive microwave remote sensing to develop LST retrieval algorithms has become a research focus.

Methods for retrieving LST information from passive microwave data can be divided into the following categories: statistical regression algorithms, physical model algorithms and machine learning algorithms. Statistical regression algorithms are widely used in the study of LST retrieval by passive microwave sensors. In the early years, McFarland et al. (1990) [17] excluded water and snow-covered areas from the study area and performed multiple regression analysis on the special sensor microwave/imager (SSM/I) brightness temperature (TB) data and the measured surface temperature in the study area. The main factor of the LST retrieval algorithm established by this author was the 37 GHz vertical polarization TB, while the other channels represented the correction factors for atmospheric water content. The error of the retrieval result ranged from 2.0 ~ 2.6 K. Njoku et al. (1999) [18] used the 37 GHz model to calculate the LST retrieval based on the analysis of the influence of atmospheric water vapor content. The model accuracy ranged between 1.5 and 3 K. Mao et al. (2007) [19] used synchronized Moderate Resolution Imaging Spectroradiometer (MODIS) LST products as ground-measured data to correspond to Advanced Microwave Scanning Radiometer-EOS (AMSR-E) TB data. The 89 GHz V polarization was found to have the highest correlation with MODIS LST, indicating that the establishment of a regression model with high-frequency vertical polarization TB data could improve the accuracy of the LST retrieval. The regression model established in the Qinghai-Tibet Plateau achieved satisfactory results with an error range of 2~3 K. These studies show that the statistical regression algorithm uses a simple method to characterize the intrinsic relationship between the TB measured by the sensor and the LST by analyzing a large amount of data. However, this method lacks a more specific physical basis and will increase the uncertainty of retrieval because it is necessary to determine the coefficients of the regression algorithm according to different land surface types.

The theoretical basis of the physical model of the passive microwave LST retrieval algorithm is the energy conservation of the Earth system, and the LST retrieval algorithm is established using the energy balance Equation. The radiative transfer Equation describes the total radiant intensity observed by an onboard microwave radiometer, including surface radiation and upward/downward atmospheric path radiation, as well as the weakening of the radiation component due to atmospheric absorption [20,21,22]. However, for each channel added by the multichannel radiative transfer Equation, an unknown number is added, and *N* frequencies always have *N*+1 unknowns, which is a problem for retrievals using in a physical model. To solve the problem of pathological retrieval, many studies [23,24,25] have assumed a linear relationship between specific radiance and vertical surface/horizontal polarization, assuming surface temperature and brightness have simple linear relationships with radiance. This relationship is then used to solve the problem of unknown radiance and thus obtain surface temperature retrievals with improved accuracy. However, these methods simplify many preconditions, such as constant atmospheric correction, absorption by surface vegetation, and neglect of atmospheric scattering effects. These assumptions affect the retrieval accuracy to some extent.

The machine learning neural network algorithm exhibits little dependence on the physical Equation of radiation transmission and can better solve the ill-conditioned retrieval problem of the physical model. Compared with the statistical regression algorithm, the machine learning neural network algorithm is better suited to solve nonlinear problems. Zurk et al. (1992) [26] transformed the soil moisture, soil temperature, and vegetation moisture data to reasonable ranges (soil moisture varying from 0 to 0.3 g/cm^3^, soil temperatures from 278 K to 313 K, and vegetation moisture between 0 and 0.18 kg/m^2^), simulated the surface temperature within that range, and used the multilayer perceptron (MLP) neural network method based on back propagation to retrieve the LST. The error of the retrieval result was 2K. Mao et al. (2009) [27] retrieved the LST from the AMSR-E TB data by using an artificial neural network algorithm and MODIS LST product. When 5 frequencies/10 channels were used, the retrieval accuracy was good. Forty-eight data sets of LST were obtained for Six FLUXNET sites (Brookings, Audubon, Fort Peck, Canaan, Black Hills, Bondville), which are located within large, mostly uniform regions (http://public.ornl.gov/ameriflux/siteselect.cfm). The comparison between AMSR-E retrieval results and ground measurements indicated that the average accuracy of the neural network algorithm was within 2K. The advantage of the neural network algorithm is that it does not need to derive physical processes for specific problems, it only needs to input a representative dataset containing many samples to train the neural network, and the trained neural network model is used for the retrieval. The above traditional machine learning neural network methods are shallow machine learning methods, and the drawback is that it is difficult to solve the problems of reaching a local optimum, overfitting and gradient diffusion. These problems occur because shallow learning algorithms perform linear and nonlinear processing on the original input data with only a small number of layers. For complex microwave radiation signals, shallow learning algorithms cannot fully learn the multilevel information in the training data. Therefore, it is necessary to use a deep learning algorithm to further optimize the microwave surface temperature retrieval algorithm. Based on the analysis of previous passive microwave LST retrieval algorithms, this paper summarizes the advantages and limitations of different algorithms and proposes the use of the convolutional neural network (CNN) method with deep learning characteristics to perform surface temperature retrieval to overcome the limitations of traditional LST retrieval algorithms. After analyzing the data samples from different band combinations or collected from different areas, we construct a model framework for the retrieval of surface temperature based on the CNN method, which improves the versatility and retrieval accuracy of the algorithm.

## 2. Study Area and Datasets

### 2.1. Study Area

**Figure 1:**
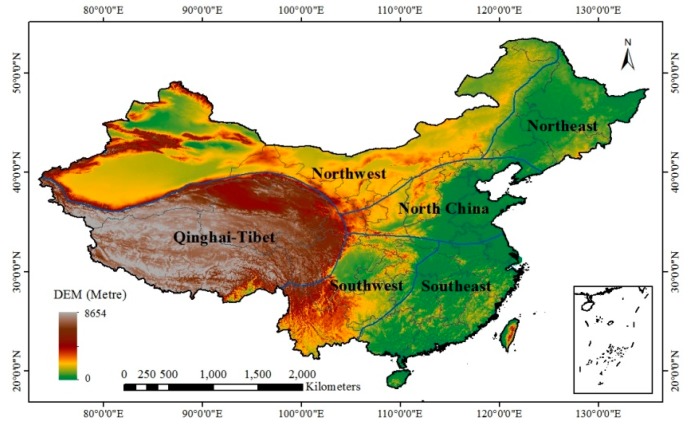
Study area.

China is located in the eastern part of the Eurasian continent, west of the Pacific Ocean, with a land area of 9.6 million km^2^. The territory ranges from 3°31′00″N to 53°33′00″N and 73°29′59″E to 135°2′30″E. The terrain of China is high in the west and low in the east. The overall terrain transforms from high to low in three steps. The main topography includes five types: plateaus, mountains, hills, basins and plains. The geographical location and diverse topography determine China’s diverse climate, making the spatial distribution pattern of rainfall "high in the southeast and low in the northwest." Because the climate types in the different parts of mainland China are distinct, China is divided into six major regions by climate type (Figure 1). The “Qinling-Huaihe” line is used as the north-south boundary line, which is divided into North China and Southeast China. The North China region is characterized by a temperate monsoon climate, while the “Qinling-Huaihe” area is mainly characterized by a subtropical monsoon climate, and the southern coastal region has a tropical monsoon climate. The southwestern region mainly includes Yunnan, Guizhou, Sichuan, and Chongqing. This region is dominated by basins and mountains, and most of the regions are characterized by a subtropical monsoon climate. The northeastern region includes Heilongjiang, Jilin, Liaoning and eastern Inner Mongolia and is mainly characterized by a temperate monsoon climate. The northwestern region is located in the hinterland of the Asian continent, including Xinjiang, Gansu, Ningxia, Shaanxi, and central and western Inner Mongolia. The climate in this region is characterized by arid or semiarid temperate continental climates. The Qinghai-Tibet Plateau region includes Qinghai Province and the Tibet Autonomous Region. As the plateau with the highest altitude in the world, the Qinghai-Tibet Plateau is widely covered by snow, ice and permafrost, and the region is mainly characterized by a plateau climate.

### 2.2. Remote Sensing Data

Advanced Microwave Scanning Radiometer 2 (AMSR2) L3 TB data are used as an independent variable for training the surface temperature retrieval algorithm in this research. The time span of the AMSR2 sensor is from July 2012 to February 2019. There are 14 horizontal/vertical polarization observation channels, which are 6.9, 7.3, 10.65, 18.7, 23.8, 36.5 and 89.0 GHz. The equatorial crossing times are at 13:30 and 1:30 local time. To generate a long-term data product using the trained CNN retrieval model, it is necessary to input a long-term sequence of passive microwave remote sensing TB data to the retrieval model, thereby retrieving the LST. Therefore, AMSR-E L3 TB data are required. The time span of the AMSR-E sensor is from July 2002 to September 2011, and the transit times are also 13:30 and 1:30 local time. The Japan Aerospace Exploration Agency (JAXA) provides AMSR2 and AMSR-E L3 TB data (http://gportal.jaxa.jp) with a resolution of 10 km.

To overcome the difficulties caused by the synchronization of the measured sample data, the MODIS LST product was used as a sample-dependent variable as an input for the CNN. The Aqua satellite is equipped with two sensors, MODIS and AMSR-E. The AMSR-E sensor has the same transit times as AMSR-2 at 13:30 (ascending) and 1:30 (descending). Therefore, MODIS LST can correspond to the TB of AMSR-2. For this purpose, the MYD11A1 data was used. Previous studies have shown that the accuracy of the product in the verification area is within 1 K under clear sky conditions [28]. To match the resolution of the AMSR2 TB pixels, the MODIS data were resampled to spatial resolutions of 1 km to 10 km, which is consistent with the spatial resolution of the AMSR2 data.

Due to the existence of the satellite launch window between the passive microwave AMER-E sensor and the AMSR2 sensor (October 2011 to June 2012), the corresponding passive microwave TB data could not be obtained for CNN retrieval. The data from the empty window period are replaced by the surface temperature product synthesized by MYD11C3 from MODIS data, and the spatial resolution is 5.6 km. The MYD11C3 data were resampled to 10 km, and the seasonal average of the surface temperature was calculated from October 2011 to June 2012 using the monthly synthetic product. MODIS products are made available to the public by the U.S. Land Processes Distributed Active Archive Center (lpdaac.usgs.gov).

### 2.3. Ground-Measured Data

**Figure 2:**
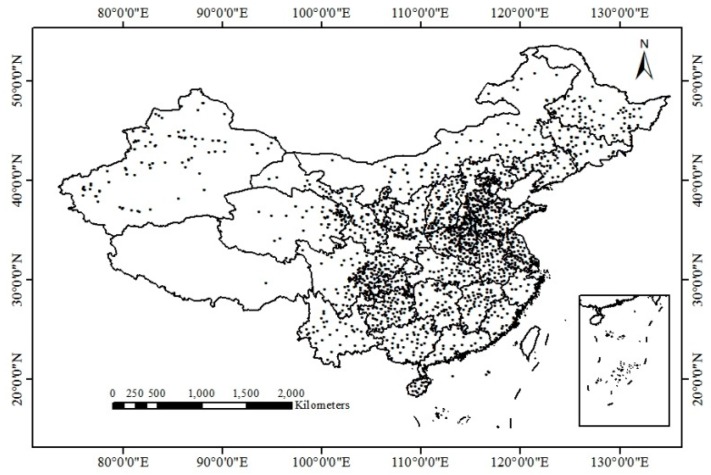
The distribution of in situ LST measurement stations in China.

The ground-measured data are used for accuracy verification. The China National Meteorological Administration (http://data.cma.cn/data/) site provides daily and hourly ground temperature monitoring data, where the ground surface temperature (GST) is measured by detectors contacting the ground surface. The time interval between measurements hourly, and the distribution of the monitoring sites is shown in Figure 2. The equatorial crossing times of the AMSR2 are 13:30 and 1:30 local time. Therefore, the average values of the LST data between 13:00 and 14:00 and between 1:00 and 2:00 from the ground monitoring site are selected. The observation times for the ground observation site data are longer, and the coverage is wider. However, there are still many sites that are missing measurements, and the values obviously deviate without quality control. Therefore, 33 sites (Table 1) with flat terrain, uniform terrain types, no obvious deviations and null values from April 2016 to October 2016 were sorted and selected, and 385 data were extracted. These data were used to verify the CNN surface temperature retrieval model.

## 3. Methods

### 3.1. Microwave Radiation Transmission Theory

A microwave radiometer measures the thermal emissions from the ground and their transfer from the ground through the atmosphere to the remote sensor on the satellite. In the microwave band, the atmospheric radiation transfer Equation for the thermal radiation balance can be described as follows [29]:

(1) $$\begin{array}{c} B_{f}\left(T_{f}\right)=\left[1-\tau_{f}\left(\theta \right)\right]\left(1-\varepsilon_{f}\left(\theta \right)\right)\tau_{f}\left(\theta \right)B_{f}\left(T_{a}^{↓}\right)\\ +\left[1-\tau_{i}\left(\theta \right)\right]B_{f}\left(T_{a}^{↑}\right)+\tau_{f}\left(\theta \right)\varepsilon_{f}\left(\theta \right)B_{f}\left(T_{s}\right) \end{array}$$

In Equation (1), *B_f_(T)* is the radiation intensity on the satellite, *T_f_* is the luminance temperature on the satellite, *τ_f_(θ)* is the transmittance of the frequency *f*, *ε_f_(θ)* is the emissivity of the frequency *f*, *B_f_(T_a_^↓^)* is the total downward radiation intensity of the atmosphere, *B_f_*(*T_a_^↑^*) is the total upward radiation intensity of the atmosphere, *B_f_(T_s_)* is the surface radiation intensity, and *T_s_* is the surface temperature. *B_f_(T)* is the Planck function:

(2) $$B_{f}\left(T\right)=\frac{2hf^{3}}{c^{2}\left(e^{\frac{hf}{kT}}-1\right)}$$

In Equation (2), *B_f_(T)* is the spectral brightness of a black body, and its units are Wm^−2^sr^−1^Hz^−1^. *T* is the absolute temperature, its units are *K*. *h* is the Planck constant 6.63×10^−34^ J. The units for the frequency *f* are Hz. *k* is the Boltzmann constant 1.38 × 10^−23^ J·K^−1^, and *c* is the speed of light of 2.992458 × 10^8^ ms^−1^. Since the approximation of the microwave band is based on Rayleigh-Jones law, Equation (2) can be simplified as:

(3) $$B_{f}\left(T\right)=\frac{2kT}{\lambda^{2}}$$

Therefore, by substituting Equation (3) into Equation (1), the following Equation can be obtained:

(4) $$T_{f}=\left(1-\tau_{f}\right)\tau_{f}\left(1-\varepsilon_{f}\right)T_{a}^{↓}+\left(1-\tau_{f}\right)T_{a}^{↑}+\tau_{f}\varepsilon_{f}T_{s}$$

The above Equation shows that the surface temperature retrieval algorithm constructed by a linear combination of TB data exhibits clear physical averaging. However, the single-channel radiative transfer Equation contains at least two unknown LSTs (*T_s_*) and surface emissivity (*ε_f_*) values, as well as atmospheric influence factors (*T_a_*). In the process of surface temperature retrieval, the microwave observation radiation signal depends on the surface soil moisture and is affected by surface roughness, attenuation and extinction characteristics of the vegetation layer, as well as surface and vegetation temperature. It is therefore difficult to explain from the physical mechanism of microwave radiation transmission that the change in TB received by a satellite is caused by the combination of one or more factors [30]. To this end, it is necessary to introduce a CNN to construct a nonlinear relationship between TB and LST.

### 3.2. CNN

**Figure 3:**
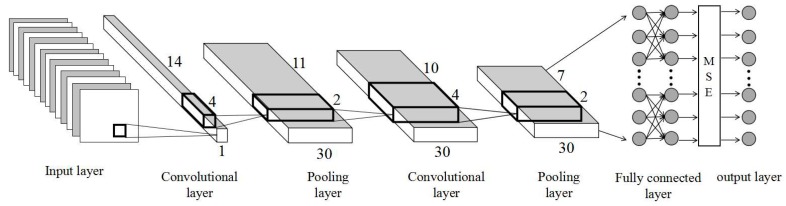
CNN architecture.

The CNN method uses a gradient descent back-propagating algorithm to train the weights in the network and achieves a global training algorithm with an improved learning rate to create a network structure with a multilayer filter in deep learning [31]. This approach reduces the complexity of the network model. In contrast to previous machine learning models, a CNN has two characteristics: local connections and weight sharing [32]. CNN algorithms use local connections to effectively extract information from data and reduce the number of parameters by sharing the weights. A typical CNN is shown in Figure 3. A CNN is mainly composed of an input layer, a convolutional layer, a pooling layer, a fully connected layer, and an output layer. Among these layers, several convolutional layers and pooled layers are alternately arranged to form multiple CNNs.

The input layer for a CNN ensures that the pixel size is uniform at 14 × 1, which means each AMSR2 brightness temperature pixels have 14 bands; that is, the size of each sample is 14 × 1, and the data are regularized. The convolutional layer is used to extract the sample features from the data and is the most central part of a CNN. Each neuron node that is input into the second convolutional layer is only part of the output from the previous layer, i.e., the output is followed by a layer of outputs for local connections [33]. To further extract the data features, the convolution layer obtains the input values of the neurons by weighted summation of the corresponding connection weights and local input values plus the offset value. The convolution operation is calculated by the following Equation (4):

(5) $$x_{j}^{l}=f\left(\underset{i\in M_{j}}{\sum }x_{i}^{l-1}\cdot k_{ij}^{l}+b_{j}^{l}\right)$$

In Equation (5), *l* is the number of layers where the convolution layer is located, *k* is a convolution kernel, *b* is a bias term, *f* is an activation function, and *M_j_* is the input characteristic data for a previous layer. The convolution kernel is a weight matrix whose size and number are manually specified. Considering that the total size of the input data is only 14, the convolution kernel size set in this study is 4 × 1, which results in a total of 30 kernels. The convolution kernel moves in steps of 1. To reduce the number of operations based on the retention of useful information and accelerate the training speed of the network, the CNN is actually equivalent to the convolution operation of the data and the convolution kernel [34]. The kernel is convolved, and then, a bias is applied to the activation function of the neuron to obtain the convolution output layer. The output of each neuron in the convolutional layer is delinearized by an activation function [35]. Commonly used activation functions include sigmoid functions, tanh functions, and rectified linear unit (ReLU) functions. Both the sigmoid function and the tanh function suffer from saturation problems, and it is easy to make the gradient disappear. The ReLU function is an unsaturated nonlinear function that can solve the problem of neural network gradient explosion and gradient disappearance and can accelerate the convergence speed of the network training [36]. Therefore, the ReLU function is used as the activation function and is calculated according to the following Equation:

(6) f(x)=max(x,0)

For ReLU, if the input is greater than 0, the output is equal to the input; otherwise, 0 is the output. With the ReLU function, the output does not tend to saturate as the input gradually increases. The CNN adds a pooling layer after each convolution operation to down-sample the output of the convolutional layer. The pooling layer can very effectively reduce the size of the matrix, thereby reducing the parameters in the last fully connected layer, accelerating the calculation speed, and preventing overfitting problems [37]. The forward propagation process of the pooling layer is also accomplished by moving a kernel function. The commonly used kernel functions have a maximum operation or an average operation. In this paper, the largest pooling method is used. The Equation is as follows:

(7) $$x_{j}^{l}=f\left(\beta_{j}^{l}max\left(x_{j}^{l-1}\right)+b_{j}^{l}\right)$$

In Equation (7), l represents the number of layers, β represents the maximum pooling coefficient, b is the bias term, and f is the activation function. The pooling layer also uses ReLU as an activation function. The pooling kernel is set to a matrix size of 2. In a CNN structure, one or two fully connected layers are connected after multiple convolution and pooling layers. The CNN described above uses two sets of convolution and pooling layers and finally a fully connected layer. Each neural node in the fully connected layer is fully connected to all neural nodes in the previous layer. The role of the fully connected layer is to integrate local information with different features in the convolutional layer and the pooled layer [38]. To maintain the stability of the CNN network, the neural network node activation function at the fully connected level also uses the ReLU function. The fully connected layer performs information transformation and calculation processing based on the high-order invariant features obtained after convolution and pooling and completes a forward propagation learning process. The CNN output layer uses the average square error (MSE) as a loss function, and the layers’ functional Equation is as follows:

(8) $$MSE\left(y,y^{\prime }\right)=\frac{\sum_{i-1}^{n}{\left(y_{i}-y_{i}^{\prime }\right)}^{2}}{n}$$

In Equation (8), *y_i_* is the target answer of the *i-th* data in a training batch, and *y_i_^’^* is the retrieval value given by the neural network. CNN optimization processes include two stages: the forward propagation stage and the backpropagation stage. The forward propagation stage is inputted from the training sample and transmitted to the output layer on a layer-by-layer basis through the convolution kernel pool [39]. The predicted value in the forward propagation stage is calculated and compared with the target value to obtain the error between the two. The backpropagation stage is used to calculate the gradient of the loss function for each parameter, and then, the gradient descent algorithm is used to update each parameter according to the gradient and learning rate so that the CNN loss function on the training data is as small as possible [40]. With massive amounts of training data, it takes a long time to calculate the loss function for all the training data. To accelerate the training process, a stochastic gradient descent (SGD) algorithm is used. The traditional SGD algorithm maintains a single parameter learning rate to update the ownership weight. The learning rate does not change during the training process. The Adam (adaptive moment estimation) algorithm was used as a gradient descent algorithm for the CNN backpropagation stage. The method was proposed by Diederik Kingma of OpenAI and Jimmy Ba of the University of Toronto in 2014 [41]. The Adam algorithm is an extension of the SGD algorithm. Compared with the traditional SGD algorithm, Adam calculates an independent adaptive parameter learning rate for different parameters by calculating the first moment estimation and the second moment estimation of the gradient. Therefore, Adam has higher computational efficiency and lower memory requirements than the traditional SGD algorithm [42]. To build a CNN, we use the Keras interface in Python and the algorithm framework provided by the TensorFlow deep learning library.

## 4. Results

To evaluate the accuracy and practicability of CNN LST retrieval, we selected the classic AMSR2 TB and MODIS LST products to establish training and test datasets. The CNN is used to evaluate and analyze the training and test datasets acquired in different ways. We perform a comprehensive CNN retrieval verification analysis using a multisource training database. Finally, we analyze the temporal and spatial variation characteristics of surface temperature in different climate regions in China over the past 15 years.

### 4.1. Analysis of CNN Retrieval Model Based on Different Channel Combinations

**Figure 4:**
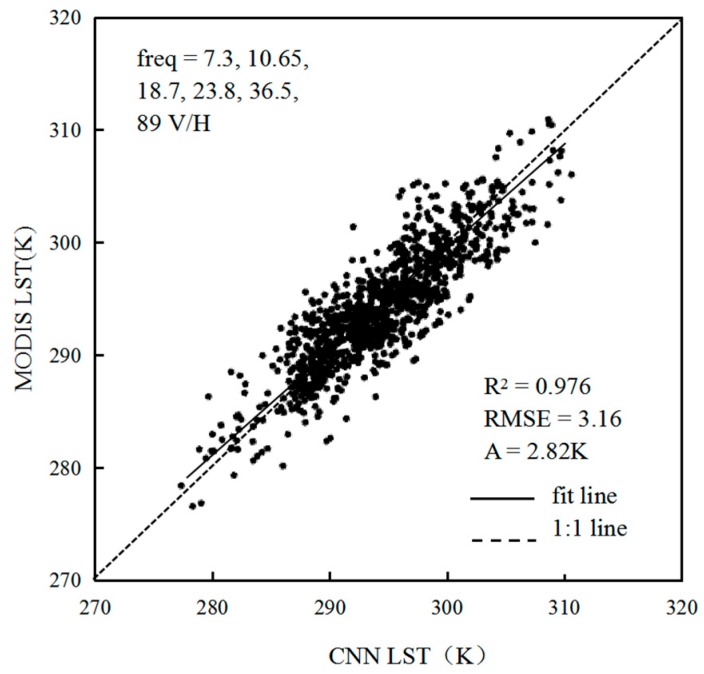
Scatterplot of CNN retrieval results and MODIS LST data.

To analyze the applicability of CNN retrieval of surface temperature, 23275 sets of TB-surface temperature samples were collected, and the dataset was randomly divided into a training dataset with 20947 samples and a test dataset with 2328 samples. The CNNs were then trained using three different channel combinations, 6.9, 7.3, 10.65, 18.7, 23.8, 36.5, and 89 GHz V/H; 7.3, 10.65, 18.7, 23.8, 36.5, and 89 GHz V/H; and 10.65, 18.7, 23.8, 36.5, and 89 GHz V/H. After continuous trials, the CNN exhibited the best retrieval after less than 3000 iterations. Table 2 shows that in the case of 3000 iterations, the first combined retrieval error correlation coefficient (R^2^) is 0.942, RMSE=3.84, and the average relative error is 3.41 K; the second combined retrieval error R^2^ is 0.976, RMSE=3.16 K, and the average relative error is 2.82 K; the third combined retrieval error R^2^ is 0.935, RMSE=3.64 K, and the average relative error is 3.29 K. As shown in Figure 4, after 6.9 GHz is excluded, the combination of six frequencies (7.3, 10.65, 18.7, 23.8, 36.5, and 89 GHz, twelve V/H channels) results in the most stable and accurate CNN retrieval model. Previous studies [27] have shown that the low-frequency data of AMSR2 are poorly related to MODIS surface temperature products. The lower the frequency is, the more different the contribution of energy from different surface layers, so the combined retrieval error decreases after the low-frequency data (e.g., 6.9 GHz) are excluded. Therefore, the combination of six frequencies (twelve V/H channels) is best suited for retrieval.

**Figure 5:**
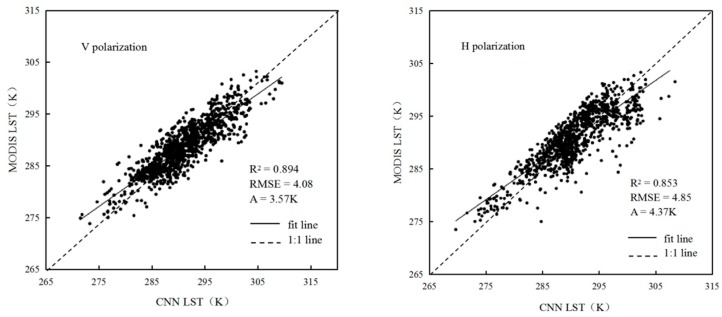
Scatterplots of CNN retrieval results (V/H) and MODIS LST data.

To analyze the error effects of the vertical and horizontal polarization modes on the CNN LST retrieval model, we divided the above datasets into two parts, vertical polarization and horizontal polarization, and then trained the CNN. With the combination of the seven channels of 6.9, 7.3, 10.65, 18.7, 23.8, 36.5, and 89 GHz, Table 2 and Figure 5 show that the CNN after 3000 iterations exhibits the best retrieval results and highest correlation. For the vertical polarization combination, the R^2^ is 0.894, the RMSE is 4.08, and the average relative error is 3.57 K. For the horizontal polarization combination, the R^2^ is 0.853, the RMSE is 4.85 K, and the average relative error is 4.37. The vertical polarization is better than the horizontal polarization, but because the CNN relies heavily on a large amount of data, training with only one polarization will reduce the number of channels. Therefore, the combination of the vertical and horizontal polarization modes is better than the combination with a single polarization mode.

### 4.2. Analysis of CNN Retrieval Model based on Different Regions 

**Figure 6:**
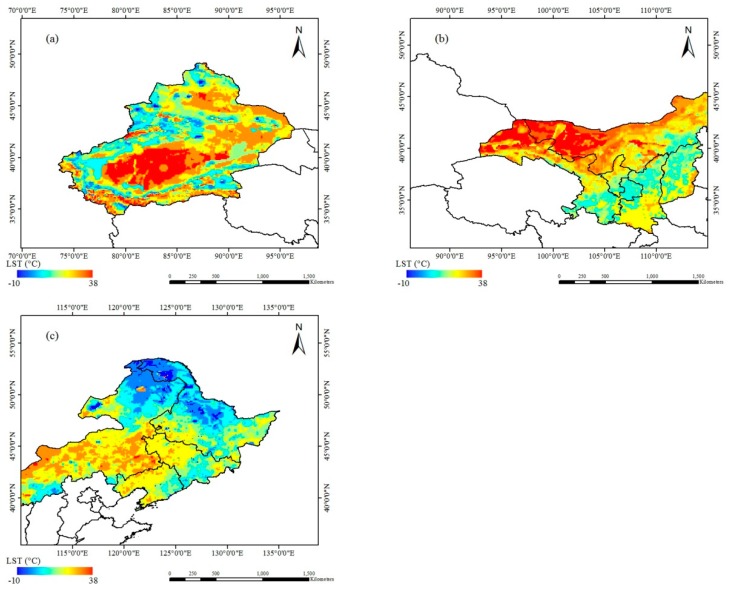
Spatial distribution of LST from the CNN results in Xinjiang (**a**), midwest Inner Mongolia (**b**) and Northeast China (**c**).

To further analyze the influence of samples from different regions on the retrieval error of the CNN, the accuracies in three regions were verified, including Xinjiang (10456 training data samples and 1134 test data samples), midwest Inner Mongolia (10598 training data samples and 1061 test data samples) and Northeast China (10327 training data samples and 1033 test data samples). According to the results of the analysis of different channel combinations, the three regions were trained with twelve V/H channels (7.3, 10.65, 18.7, 23.8, 36.5, 89 GHz V/H). The results of the CNN retrieval of the surface temperature for the test sample data are shown in Table 3. In Xinjiang, R^2^ = 0.981, RMSE = 2.84 K, and the average relative error is 2.61 K. In midwest Inner Mongolia, R^2^ = 0.979, RMSE = 3.16 K, and the average relative error is 2.82 K. In Northeast China, R^2^ = 0.969, RMSE = 3.25 K, and the average relative error is 3.03 K. These results indicate that the CNN surface temperature retrieval model has the highest accuracy in Xinjiang. The retrieval results of the three regions (Figure 6) indicate that the LST from the CNN retrieval model can adequately represent the actual overall spatial variation, indicating that a CNN model can be applied to surface temperature retrieval, especially over large areas of bare land (such as Xinjiang), where the model precision is high.

### 4.3. Analysis of the CNN Retrieval model Based on Different Regions.

Ground verification is the key to the practical application of the LST retrieval model. At present, the actual accuracy verification of the LST retrieval model is a point of difficulty in the research on retrieval methods. The main reason for this difficulty is that the ground-point measured data and the large-scale remote sensing retrieval results cannot be accurately combined on the spatial scale of expression. Ground verification is very important for the practical application and promotion of the method. Therefore, it is necessary to verify the analysis by comparing the LST data from a measurement site with the CNN retrieval results. The AMSR2 TB image corresponding to the time of the trained CNN is input to retrieve the LST. Then, the retrieval result pixel is extracted according to the latitude and longitude of the station. Finally, the extracted data are compared with the ground-measured data for verification.

**Figure 7:**
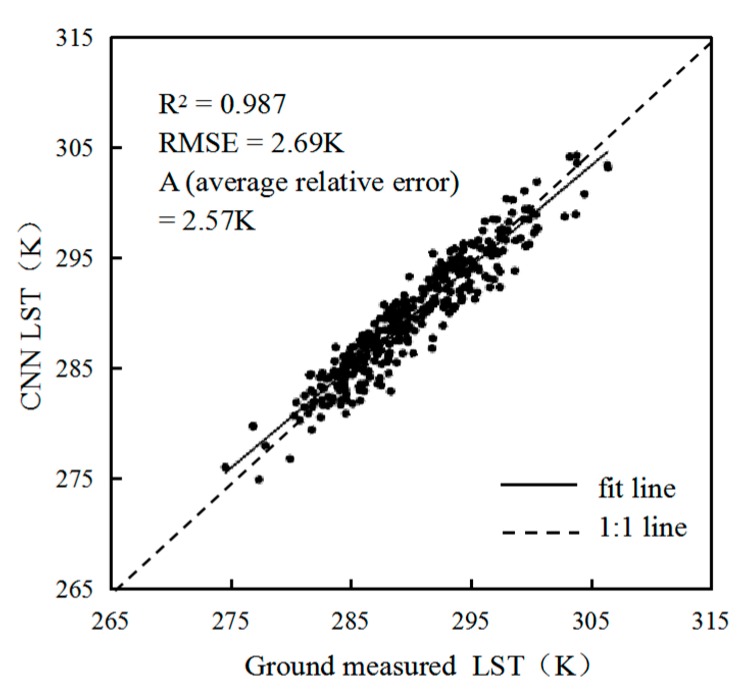
Scatterplot of CNN retrieval results and ground-measured data.

The results are shown in Figure 7. The average relative error between the value retrieved by the CNN model and the ground-measured data is 2.57 K, and the R^2^ is 0.987, RMSE = 2.69 K. Although the data representation is as close as possible to the sample collection, the CNN retrieval results are somewhat lower than the site data, which may be related to the MODIS LST product being one of the sources of the dependent variable sample. Because the TB values from passive microwave and thermal infrared data are somewhat different, the passive microwave TB is deeper than the thermal infrared TB. In addition, it is difficult to accurately verify the surface temperature on the ground. Ground-based observation sites generally use measurements from gridded points, which is different from the large-area surface measurements of microwave radiometers. Therefore, this difference requires an appropriate correction of the retrieval results in the actual CNN retrieval. When selecting the CNN training sample data, the synchronization problem between the passive microwave TB and the ground observation data is overcome as much as possible, and some pixels with relatively large error are removed. These pixels may be affected by large areas of rainfall or other factors. The advantage of a CNN is that it increases the retrieval accuracy by adding enough ground training data with high precision to the MODIS LST product, which is applicable to more complex surface types.

**Figure 8:**
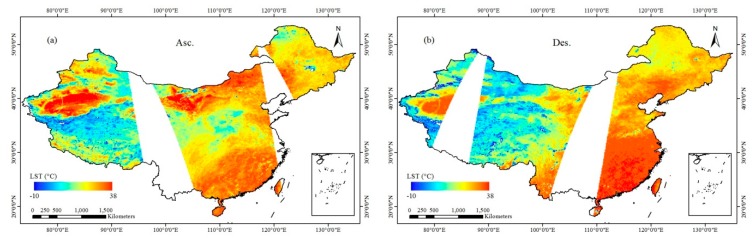
LST distribution of CNN retrieval results in China on August 13, 2016 (**a**) Asc.; (**b**) Des.

To further analyze the retrieval results, the CNN LST retrieval model was applied to the ascending and descending AMSR2 TB data on August 13, 2016. The LST values of China were inferred, and the spatial variations were generated (Figure 8). Figure 8 shows that the retrieval results are basically consistent with the dry and wet distribution in China. The LST values around the coast, Yangtze River, Yellow River and some large lakes are low, which is consistent with the actual situation. The LST of the CNN retrieval is consistent with the spatial distribution characteristics of surface temperature in summer in China. The retrieval results of the ascending pass (Figure 8a) show that during the day (13:30), the surface temperatures of the Badain Jaran Desert, Tengger Desert, and Taklimakan Desert in Xinjiang are the highest. The temperature south of the Yangtze River is generally higher than that in North China and Northeast China. In the daytime LST retrieval results, Shanxi and Shaanxi Provinces have small pixel values, mainly because the input TB pixels are affected by rain during the day. The results of descending pass in central and western Inner Mongolia at night (1:30) (Figure 8b) show that the surface temperatures of the Badain Jaran Desert, Tengger Desert, and Taklimakan Desert in Xinjiang are low, which is in line with the geographical characteristics of the abovementioned day and night temperature differences. The surface temperatures in the Qinghai-Tibet Plateau are the lowest during the day and night, which is consistent with the characteristics of the plateau climate. Some of the pixel values are low due to lakes and alpine ice. In general, the CNN surface temperature retrieval algorithm based on passive microwave remote sensing performs well. Of course, in the future, it will be necessary to supplement more training sample data with high precision over representative regions to further improve the retrieval accuracy of the algorithm.

### 4.4. Spatiotemporal Variations in Summer Soil Moisture in China.

The temporal and spatial changes are analyzed at the seasonal scale in this research. First, according to meteorology, spring is from March to May, summer is from June to August, autumn is September to November, and winter is from December to February of the next year. The LST dataset is derived from the retrieval results obtained from the CNN model trained in the previous section. The precision of the model is verified, and the retrieval accuracy is high. The TB data of the ascending and descending passes are input to the CNN retrieval model, and then the average of the diurnal results is calculated to obtain the daily average LST. Finally, the seasonal average is calculated from the daily average value.

**Figure 9:**
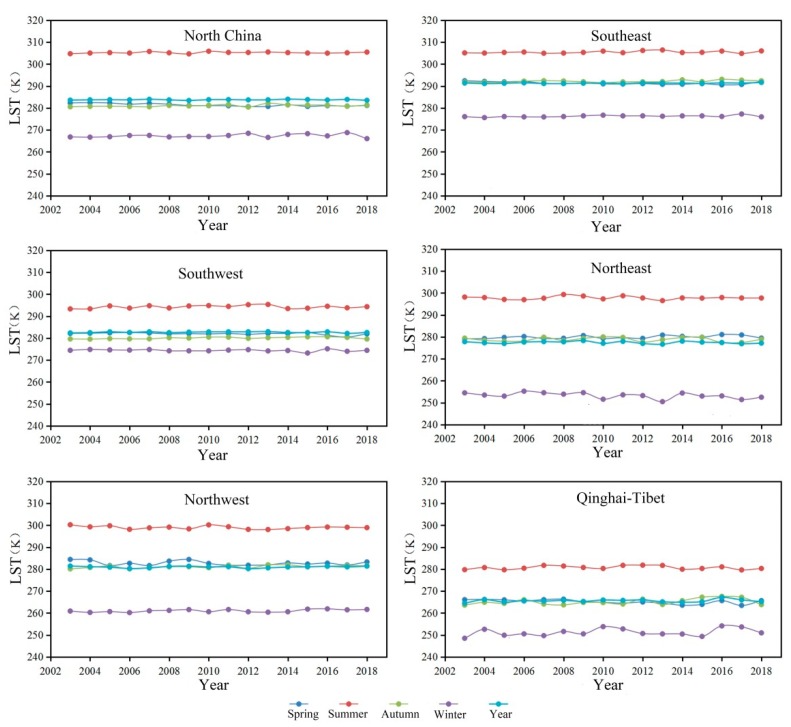
Variations in annual average LST from 2003 to 2018.

Based on the seasonal average of LST, the annual variations in LST in the six major regions of China were calculated by means of raster pixel subregional statistics (Figure 9). The change in LST from 2003 to 2018 in China was small. There were significant seasonal differences in the magnitude of the change, with the spring and autumn averages being close to the annual average, but the summer and winter averages are the highest and lowest, respectively. Over the past 15 years, the areas with large fluctuations in LST were mainly in Northeast China in the winter and the Qinghai-Tibet Plateau. The average winter LST in Northeast China was 253.3 K (−19.9 °C), which exhibits fluctuations, and the lowest value was reached in 2013 (250.5 K, i.e., −22.7 °C). The annual LST in the Qinghai-Tibet region averaged 251.2 K (−21.9 °C), and the maximum values in 2004, 2010 and 2016 were 252.6 K, 253.8 K and 254.1 K, respectively, corresponding to −20.6 °C, −19.6 °C and 19.6 °C. Compared with other regions, the LST in the Qinghai-Tibet region was the lowest in all four seasons. The summer average in the Qinghai-Tibet region was 280 K (7 °C), which was mainly affected by the geographical features of the plateau mountains in the Qinghai-Tibet Plateau. In contrast to the Qinghai-Tibet Plateau, the highest LST was found in the southeast. The annual average was 291.3 K (18.1 °C), the average in summer was 305.4 K (32.3 °C), and the average in winter was 276.2 K (3.1 °C).

After the seasonal average of LST data was calculated, least squares linear regression analysis was used to analyze the LST trends from 2003 to 2018 in China. 

The slope reflects the trend of the variable during the study period, and a slope greater than 0 indicates that the variable is increasing; otherwise, it is decreasing. To effectively analyze the significance level of the change trend, the slope of each season was subjected to an F test. At the significance test levels of 0.01 and 0.1, the results were divided into three categories: basically unchanged, significant change, and very significant change. Using the grid calculator in ArcGIS, the classification result is multiplied by the positive and negative slope values, and the five types of changes are significantly decreasing, significantly decreasing, substantially unchanged, significantly increasing, and very significantly increasing.

**Figure 10:**
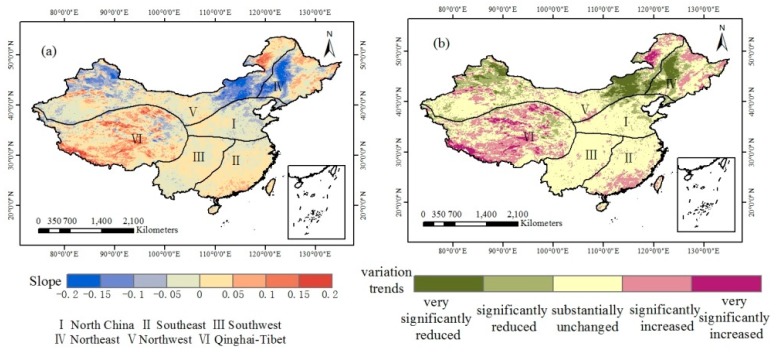
Annual average LST changes from 2003 to 2018.

For the regional average surface temperature from 2003-2018 in China, the linear change slope, correlation coefficient and trend classification are calculated. Figure 10 shows that the slope of the annual average LST value has been concentrated in the range of (−0.015, 0.015) over the past 15 years. Table 4 shows that the area where the LST remains basically unchanged accounts for a large proportion, reaching 59.33% of the area, corresponding to 5.69 million km^2^. The area where the LST has risen significantly covers 19.73% of the country, with an area of 1.89 million km^2^, and these trends are mainly distributed in Guangdong Province, Heilongjiang Province and parts of the Qinghai-Tibet Plateau. The significantly decreasing area accounts for 13.14% of the country, with an area of 1.26 million km^2^, and these trends are mainly distributed in Northwest and Northeast China. The region with a very significant reduction accounts for 5.13% of the country, and these trends are scattered in eastern Inner Mongolia, Sichuan Province and Guizhou Province. The areas that are significantly rising are concentrated in the southern part of the Qinghai-Tibet Plateau and the Hulunbuir grassland in Inner Mongolia, accounting for only 1.30% of the country.

**Figure 11:**
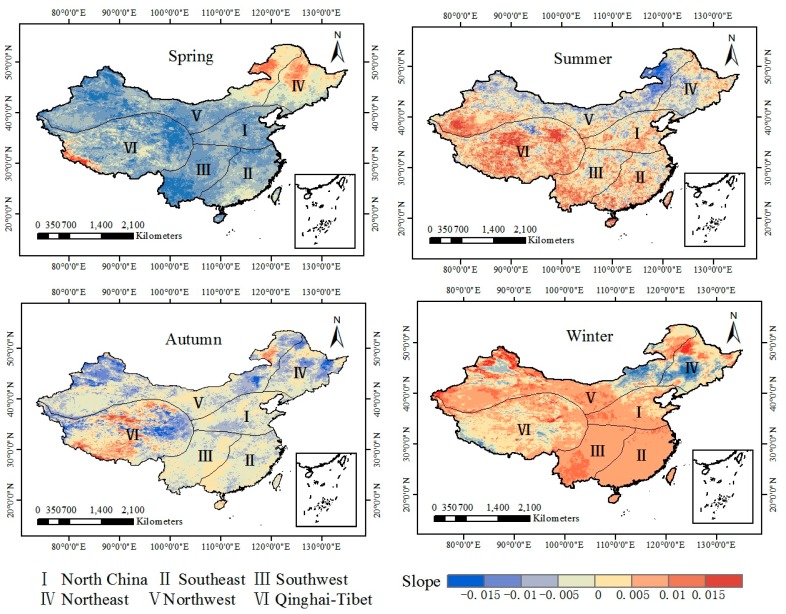
Slopes of the seasonal average LST from 2003 to 2018.

To further evaluate the trend of the annual spatial variations in LST in various seasons, we calculated the slope (Figure 11) for the pixel-by-pixel LST data of China from 2003 to 2018. The slope ranges between -0.015 and 0.015 in the four seasons, indicating that the average LST in China over the past 15 years exhibits a small change. In most areas in spring, the LST showed a downward trend (slope s < 0, correlation coefficient r < 0, which are the blue and green parts in the figure, respectively), and a small part (e.g., Northeast China and the west of the Qinghai-Tibet Plateau) showed an upward trend (s > 0, which is the red part of the figure). In addition to Inner Mongolia and northern Xinjiang, the summer LST exhibits an overall upward trend, with the most obvious trends in the Tarim Basin and the Kunlun Mountains (slope s < ×0.015). In autumn, the area where the slope is less than 0 is widely distributed, indicating that the LST in autumn generally shows a downward trend. The declining trends in Heilongjiang, central Inner Mongolia, Qinghai and northern Xinjiang are obvious. In winter, LST generally shows an increasing trend. The slopes in North China, Southeast China and Southwest China were all greater than 0, and a small number of regions show downward trends, such as central Inner Mongolia, Liaoning Province and Jilin Province.

**Figure 12:**
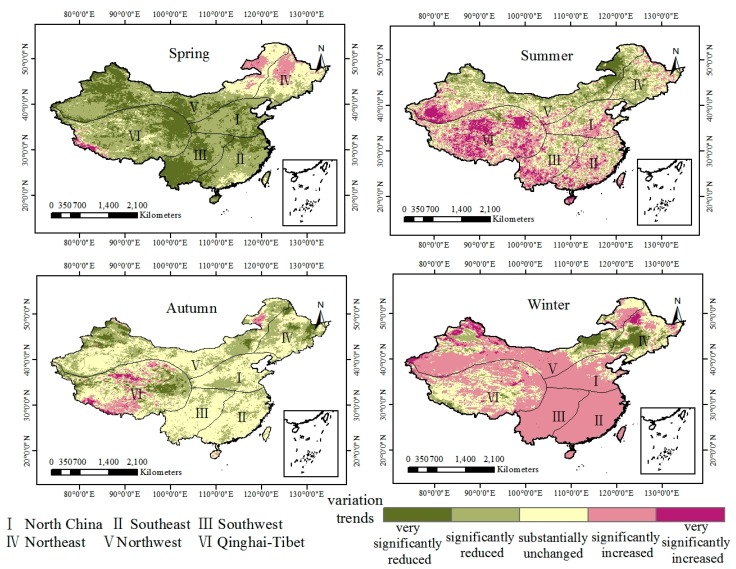
Spatiotemporal variations in seasonal average LST from 2003 to 2018.

The seasonal average LST slopes in China from 2003-2018 were classified (Figure 12), and the area ratios of the seasonal trend types in spring, summer, autumn and winter were calculated (Table 5). The results indicate that the LST in China in spring has generally decreased over the past 15 years. In southern China, North China, Northwest China, and the Qinghai-Tibet Plateau, there were significant decreases. The areas with significant decreases and extremely significant decreases covered 4.78 million km^2^ and 2.94 million km^2^, respectively, accounting for 49.77% and 30.69% of the national area, respectively. The regions with a significant increase in LST in spring accounted for only 0.39% of the country, which were mainly in the southwestern region, Gansu Province, Qinghai Province and northern Xinjiang, while significant increases occurred in the northeastern region, northeastern Inner Mongolia, and the border of the Qinghai-Tibet Plateau. In contrast to spring, the winter surface temperatures in southern China, North China, Northwest China and the Qinghai-Tibet Plateau showed significant increasing trends, with significant increases over 6.19 million km^2^, accounting for 64.46% of the country. The regions with downward trends in winter accounted for only 10.81% of the country, which were mainly distributed in Jilin, Liaoning, central Inner Mongolia and northern Xinjiang. In the summer, 10.14% of the regions showed very significant increases in LST, with an area of 0.97 million km^2^, which was the highest of the four seasons. The areas that significantly increased were mainly distributed in the Tarim Basin, the Qaidam Basin, Yunnan Province, and the original region of the rivers in Qinghai Province. The area where the LST in the autumn remained basically unchanged covered 54.35% of the country, corresponding to an area of 5.21 million km^2^, which is the highest of the four seasons, indicating that the LST changes in China were the most stable in autumn.

## 5. Discussion and Conclusions

This research applies the deep learning CNN method to the passive microwave LST retrieval problem. The retrieval accuracy of deep learning CNNs mainly depends on the training and test datasets. However, the spatial resolution of passive microwave data is relatively low, and it is difficult to obtain ground-measured data that are synchronized with satellite data. Therefore, we chose to use the MODIS surface temperature products as a reference to obtain ground synchronization data, thus overcoming the problem of synchronous ground observation data. The AMSR2 TB data and MODIS LST data were randomly divided into training and test datasets; a CNN was constructed to simulate the passive microwave radiation transmission process, and the LST was inverted. The main conclusions are as follows:

First, the combination of twelve V/H channels of 7.3, 10.65, 18.7, 23.8, 36.5, and 89 GHz results in the most stable and accurate CNN retrieval model. Vertical polarization is better than horizontal polarization. However, because the CNN rely heavily on large amounts of data, training with only one polarization reduces the number of channels by half; thus, the combination of both vertical and horizontal polarizations is better than the use of a single polarization.

Second, the influence of different regional samples on the retrieval errors of the CNNs was further analyzed, and the training results in Xinjiang, Inner Mongolia, Central and Western China were verified. The results showed that in Xinjiang, the R^2^ = 0.981, RMSE = 2.84 K, and the average relative error is 2.61 K. These results indicate that the CNN surface temperature retrieval model exhibits the highest accuracy in Xinjiang, indicating that the CNN surface temperature retrieval exhibits the highest accuracy over large areas of bare land.

Moreover, the fitted line of the CNN retrieval data and ground station data is generally close to 1:1, indicating that the CNN surface temperature retrieval can maintain the overall spatial variations presented by the actual values. This result shows that the CNN method can be applied to LST retrieval. After ground precision verification, the R^2^ = 0.987, RMSE = 2.69 K, and the average relative error is 2.57 K, which indicates that the accuracy of the CNN surface temperature retrieval algorithm is high.

Finally, according to retrieval results based on a long time series, we analyze the temporal and spatial variations in surface temperature in different climate regions in China over the past 15 years. Over the past 15 years, the average surface temperature in China has generally changed slightly. From a spatial perspective, the surface temperatures are generally decreasing in spring, and most areas of China (including the southern, northern, northwest, and Qinghai-Tibet Plateau regions) exhibit significantly decreasing trends. In contrast to the temperatures in spring, most of the winter surface temperatures in China show a significant increasing trend, indicating that the surface temperatures in winter and spring in China have changed in the same regions over the past 15 years, but the changes are generally small.

Because passive microwave sensors are able to penetrate the atmosphere, microwave radiation energy comes from different levels than visible light and thermal infrared radiation. Therefore, the LST retrieval results of passive microwave sensors are different from other ground reanalysis data and thermal infrared LST products. In addition, because of the inevitable mixed pixel problem of satellite remote sensing, the spatial resolution of passive microwave remote sensing is currently low. When the microwave radiometer on the satellite passes over the study area, the microwave radiation is instantaneously measured. Therefore, it is ver difficult to obtain the measured data at the surface at this exact moment. The ground conditions must be balanced with the corresponding atmospheric conditions, topography and land cover types. This requirement results in difficulty in verifying the accuracy of remote sensing surface parameter retrieval. With the further improvement of the surface measurement network in the future by adding sufficient ground high-precision training data, the deep learning advantages of CNNs can be used to adapt to more complex surface types and further improve the retrieval accuracy of the model.

## Figures and Tables

**Table 1:** Site information.

Num.	Name of site	Province	Longitude (E)	Latitude (N)
1	Changbai Mountain	Jilin	128.10	42.40
2	Dinghu Mountain	Guangdong	112.53	23.17
3	Dinghu Mountain	Sichuan	102.00	29.58
4	Guantan	Gansu	100.25	38.53
5	Zhaoxian	Hebei	114.93	37.80
6	Laoshan	Heilongjiang	127.57	45.33
7	Mulun	Yunnan	101.27	21.93
8	Qianyanzhou	Jiangxi	115.06	26.74
9	Taihuyuan	Zhejiang	119.34	30.18
10	Xiaolangdi	Henan	112.47	35.02
11	Xishuangbanna	Yunnan	101.27	21.93
12	Yveyang	Hunan	112.51	29.31
13	Hobq	Inner Mongolia	108.69	40.54
14	Ahrou	Qinghai	100.46	38.04
15	Changling	Jilin	123.50	44.58
16	Duolun	Inner Mongolia	116.28	42.05
17	Fukang	Xinjiang	87.93	44.28
18	Haibei	Qinghai	101.30	37.60
19	Sonid Left Banner	Inner Mongolia	113.57	44.08
20	Siziwang Banner	Inner Mongolia	119.90	41.79
21	Tianjun	Qinghai	98.32	38.42
22	Tongyu	Jilin	122.52	44.59
23	Xilin Hot	Inner Mongolia	116.33	44.13
24	Xilingol	Inner Mongolia	116.67	43.55
25	Dingxi	Gansu	104.58	35.55
26	Guantao	Hebei	115.13	36.52
27	Jinzhou	Liaoning	121.20	41.15
28	Luancheng	Hebei	114.67	37.83
29	Ulan Usu	Xinjiang	85.82	44.28
30	Weishan	Shandong	116.05	36.65
31	Wuwei	Gansu	102.85	37.87
32	Panjin	Liaoning	121.90	41.14
33	Yunxiao	Fujian	117.42	23.92

**Table 2:** Errors under different channel combinations.

Epoch Size	6.9, 7.3, 10.65, 18.7, 23.8, 36.5, 89 V/H			7.3, 10.65, 18.7, 23.8, 36.5, 89 V/H			10.65, 18.7, 23.8, 36.5, 89 V/H		
	R^2^	RMSE	A (K)	R^2^	RMSE	A (K)	R^2^	RMSE	A (K)
1500	0.881	3.74	3.41	0.937	3.54	3.19	0.905	4.37	4.16
2000	0.876	3.89	3.57	0.961	3.42	2.96	0.914	3.83	3.53
2500	0.883	4.27	3.73	0.958	3.37	2.94	0.927	3.82	3.49
3000	0.924	3.84	3.34	0.976	3.16	2.82	0.935	3.64	3.29
3500	0913	3.67	3.32	0.924	3.67	3.28	0.946	3.57	3.18

Note: A: average relative error.

**Table 3:** Errors resulting from different channel combinations in Xinjiang, Inner Mongolia and Northeast China.

Epoch Size	a 7.3, 10.65, 18.7, 23.8, 36.5, 89 V/H			b 7.3, 10.65, 18.7, 23.8, 36.5, 89 V/H			c 7.3, 10.65, 18.7, 23.8, 36.5, 89 V/H		
	R^2^	RMSE	A (K)	R^2^	RMSE	A (K)	R^2^	RMSE	A (K)
1500	0.937	3.54	3.19	0.925	3.81	3.49	0.928	3.91	3.28
2000	0.961	3.42	2.96	0.967	3.47	3.06	0.957	3.78	2.99
2500	0.978	3.37	2.94	0.958	3.39	3.04	0.958	3.57	3.06
3000	0.981	2.84	2.61	0.979	3.16	2.82	0.969	3.25	3.03
3500	0.924	3.67	3.28	0.936	3.67	3.28	0.917	4.02	3.88

Note: A: average relative error

**Table 4:** Statistics of annual average LST of different variation levels.

	Very Significantly Decreasing	Significantly Decreasing	Basically Unchanged	Significantly Increasing	Very Significantly Increasing
Square (million km^2^)	0.49	1.26	5.69	1.89	0.256
Percentage (%)	5.13	13.14	59.33	19.73	2.68

**Table 5:** Statistics of seasonal average LST of different variation levels.

		Very Significantly Decreasing	Significantly Decreasing	Basically Unchanged	Significantly Increasing	Very Significantly Increasing
Spring	Square (million km^2^)	2.94	4.78	1.34	0.5	3.75
	Percentage (%)	30.69	49.77	13.97	5.19	0.39
Summer	Square (million km^2^)	0.42	2.2	3.28	2.72	0.97
	Percentage (%)	4.39	22.96	34.14	28.36	10.14
Autumn	Square (million km^2^)	0.6	3.08	5.21	0.6	10.92
	Percentage (%)	6.21	32.08	54.35	6.22	1.14
Winter	Square (million km^2^)	0.31	0.73	2.09	6.19	28.81
	Percentage (%)	3.23	7.58	21.74	64.46	3.00
